# Wakefulness

**Published:** 1884-02

**Authors:** 


WAKEFULNESS. Women require more sleep than men, other things being equal, the nervous system being more active in blowing up their husbands, studying how to marry off their daughters, setting traps for rich widowers, and gosling young gents whose dads are millionaires. Few persons after fifty can sleep longer than seven hours, unless they are hard out door workers; healthy children under ten ought to have ten hours for slee ; school girls from 12 to 18 ought to sleep at least nine hours But from various causes there is a great difference in the amount of sleep required by different persons, hence each should observe for himself how much sleep he requires and arrange to give nature that much every night; if unusual exertions are made any day, sleep longer the ight following. If kept up several hours later than usual, on chance occasions, arrange not to be disturbed in any way next morning, and when nature wakes up, get up and do not sleep any during the day, but go to bed at the regular hour and the increased soundness of sleep for that night will make up for the loss.

If you cannot go to sleep when you first go to bed, give orders to be waked up at daylight, get up promptly, do not sleep a wink during the day, go to bed at your regular time, with directions to be waked up as before; in a week you will find that you can go to sleep promptly, but then be careful to get up as soon as you wake in the mornings, thus you will soon find out how much sleep your system requires, and act accordingly; always avoiding sleeping in the day time; for if you require seven hours sle p, and spend that much in sleep at night, whatever time you spend in sleep during the day must be deducted from that seven hours, or you will soon become wakeful again. If you wake up in the night, either go to bed two or three hours later or when you

**wake, get up, even if it be but one o'clock in the morning, and do not sleep a moment until your regular hour for going to bed; and if you go to bed regularly, get up as soon as you wake, and do not sleep in the day time, you will find out in less than a week how much sleep you require, then act accordingly. Nature loves regularity, and the four hours sleep from ten to two, is worth six hours after twelve o'clock. The great rule is, retire at a regular early hour and get up always as soon as you wake, if it is daylight. If persons have force of will enough to keep from going to sleep a second time, it is greatly better to remain in bed ten or fifteen minutes after waking up, to think about it, and enjoy the resting of that kind of feeling of pleasurable tiredness which comes over us on waking, especially if we have taken more exercise than usual the previous day, or have been kept up later.**

